# Bronchioloalveolar Carcinoma in a Striped Dolphin (*Stenella coeruleoalba*) Stranded on Thyrrhenian Sea Coast

**DOI:** 10.3390/vetsci12111061

**Published:** 2025-11-05

**Authors:** Maria Dimatteo, Maria Oliviero, Marianna D’amore, Luigia Contaldo, Giuseppe Lucifora, Stefania Giglio, Giovanna Fusco, Barbara degli Uberti

**Affiliations:** 1 Istituto Zooprofilattico Sperimentale del Mezzogiorno, 80055 Portici, Italy; maria.dimatteo@izsmportici.it (M.D.); marianna.damore@izsmportici.it (M.D.); luigia.contaldo@izsmportici.it (L.C.); giuseppe.lucifora@izsmportici.it (G.L.); giovanna.fusco@izsmportici.it (G.F.); barbara.degliuberti@izsmportici.it (B.d.U.); 2 Associazione M. A. R. E. Calabria, 88060 Montepaone, Italy; associazionemarecalabria@gmail.com

**Keywords:** cetaceans, striped dolphin, lung cancer, carcinoma, metastasis

## Abstract

**Simple Summary:**

The available data on neoplasms in aquatic species, like cetaceans, come mainly from incidental findings during autopsies. Indeed, our knowledge about cancer prevalence is generally understated when it is compared to the neoplastic studies in pets. In this study we discuss macroscopical, histopathological, and immunohistochemical features of a lung cancer with lymph node metastasis in a striped dolphin (*Stenella coeruleoalba*)—a species where it appears to not previously have been recorded yet. This could represent the first report in this species and contribute to the limited existing records of neoplasm in these animals.

**Abstract:**

An adult male striped dolphin was found stranded on the Tyrrhenian coast of Belvedere Marittimo (Cosenza, Italy). The animal was multi-parasitized and post-mortem examination revealed a focal extensive white soft lesion with poorly defined margins on the caudal portion of the left lung. The tributary lymph node had similar multifocal lesions in the cortex and medulla. Histological analysis exhibited the presence of lung carcinoma with lymph node metastasis. Immunohistochemical examination allowed the characterization of the epithelial neoplasm as a bronchioloalveolar carcinoma. It appears to be the first case of lung cancer recorded in this species.

## 1. Introduction

In the literature, the data available on the neoplasms of aquatic mammals were not so common as those collected for terrestrial mammals or for domestic animals. In fact, these were often occasional findings from when these animals were found stranded along the coasts, most often due to impact with human activities. According to a study conducted on cetacean species stranded along Ligurian coasts (Mediterranean Sea) between 2007 and 2014, the most frequent causes of death were related to infectious diseases, while in only 8% of the cases, the death was related to anthropic activities such as fishing that could cause traumatic lethal injuries in cetaceans [1]. In particular, a high incidence of infectious diseases including viral, bacterial, and parasitic origin were recorded as the primary cause of death in the striped dolphin (*Stenella coeruleoalba*). Among the main emerging etiological agents, cetacean Morbillivirus (CeMv) played a central role in recent years. According to a recent study, CeMv represented the most common pathogen detected in cetaceans with 36.5% of tested animals testing positive [2]. This virus was responsible for serious morbid conditions with immunosuppression that affected this species in the Mediterranean Sea where it led to the last epidemics during 2006–2008 and 2010-2011 along the Canary Islands [3]. In the last massive cetacean mortality event in 2013, CeMv also seemed to play a crucial role in the outbreak [2,4].

However, neoplastic processes were rarely mentioned among the causes of death in cetaceans. It is widely recognized that there is an elevated number of neoplasms reported in California sea lions (*Zalophus californianus*) and beluga whales (*Delphinapterus leucas*) of the St. Lawrence Estuary in North America with a mortality rate attributed to cancer equal to 1.9% and a tumor incidence in the population of 2.5% including white-sided dolphins (*Lagenorhynchus obliquidens*) [5]. Among marine mammals, odontocetes include species with the highest number of reported cases of neoplasia in different geographical areas. The most representative neoplasms were epithelial tumors of the gastrointestinal tract, in particular, adenocarcinoma and gastric papilloma. In fact, these species have shown a particular predisposition. After that, genital, mainly fibroleiomyomas, and integument tumors occupied the second position with an incidence of 24% among cetaceans’ tumors, according to a less recent review [6]. As reported in the summary of a 2006 review [7], the most frequent tumors observed in bottlenose dolphins (*Tursiops truncatus*) were squamous cell carcinoma (SCC), described in different organs including the lung, followed by adenocarcinoma (ACA) and hematopoietic tumors such as lymphoma and myeloid leukemia [8]. In a retrospective review of 2022 on seven adult harbour poirposes (*Phocoena phocoena*) from the North and Baltic seas, the first reports of an adenocarcinoma in the liver, a testicular Sertoli cell tumor, and adrenocortical adenomas were described in the mentioned species [9]. In an adult female common dolphin (*Delphinus delphis*) stranded in the Canary Islands, a T-cell lymphoma affecting the thalamus was reported [10], while a high-grade astrocytoma was described in an Atlantic spotted dolphin (*Stenella frontalis*) [11].

In the striped dolphin, a few cases of cancer were described including a case of cutaneous SCC [7,12], a primary neuroectodermal tumor [13], and a case of glioblastoma in the central nervous system [14] but there was no evidence of a pulmonary tumor reported in this species.

The only other reports of primary lung tumors in cetaceans include SCC in an Amazon river dolphin (*Inia geoffrensis*) [6], a carcinoma with disseminated metastases to the thoracic lymph nodes and adrenal gland in a long-finned pilot whale (*Globicephala melas*) [15], and a poorly differentiated pulmonary SCC with thoracic lymph node and renal metastases in an Atlantic bottlenose dolphin [16].

In this paper we described the first case of lung carcinoma metastasized to the caudal mediastinal lymph node reported in a striped dolphin.

## 2. Materials and Methods

In 2019 an adult male striped dolphin was found stranded along the Thyrrhenian coast of Belvedere Marittimo (Cosenza, Italy). Post-mortem examination was performed according to protocol [17], and the animal cadaver was fresh at the time of necropsy.

Kidney, lung, mediastinal lymph node, peritoneum, stomach, and testicle were collected for histological examination. Tissue fragments were collected from affected and adjacent unaffected areas and fixed in 10% neutral buffered formalin, routinely processed and embedded in paraffin, microsectioned at 3–4 µm, and Hematoxylin-Eosin stained with autostainer XL (ST Infinity H&E staining System kit, Leica Biosystems, Buccinasco, Italy). Immunohistochemical (IHC) examination was performed on the lung and mediastinal lymph node sections using a Leica Bond III automated immunostainer (Leica Biosystems). An antibody panel was applied, including vimentin (clone V9), high molecular weight cytokeratin (clone 34βE12), cytokeratin 7 (clone RN7), and pan-cytokeratin (AE1/AE3). All antibodies were prediluted and ready to use (Leica Biosystems, Newcastle Ltd, Newcastle upon Tyne, England). The IHC protocol was carried out according to the manufacturer’s instructions. They were all prediluted and ready to use (Leica Biosystems, Newcastle Ltd). Immunohistochemical protocol followed manufacturer’s instructions. The slides were then digitalized with Panoramic 250 FLASH III scanner to capture photomicrographs.

## 3. Results

The striped dolphin measured 2.1 m in length and weighed 71.32 kg. On macroscopic examination, the animal showed a poor nutritional status underlined by the gelatinous atrophy of the blubber and showed several wounds deriving from anthropic activity. Within the subcutis of the caudal fin there were multiple white shiny cysts measuring approximately 1.5 cm, consistent with Phyllobothrium spp. cysts. Additionally, numerous cysts of Monorygma spp. were observed in the subperitoneal muscle. Cestode larvae were observed within hepatic and pancreatic ducts. The lungs were heavy and exuded serous fluid, and there were several adult nematodes.

**Figure 1:**
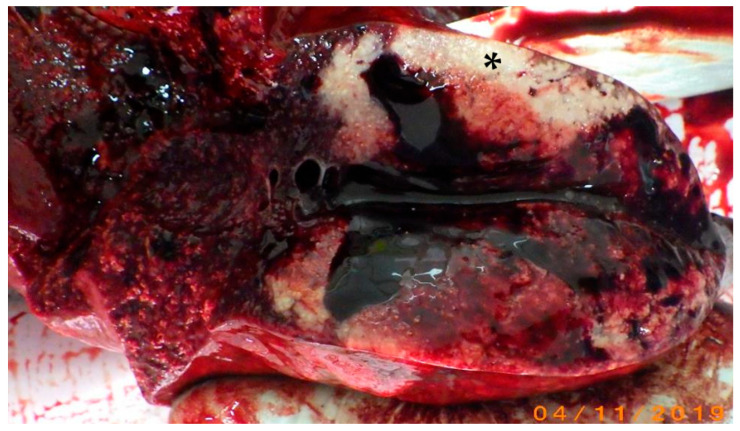
Gross lung finding in a *Stenella coeruleoalba* showing a white soft focally extensive parenchymal lesion (asterisk).

Within the caudal aspect of the left lung lobe there was a focal soft ovoid white mass, measuring 7 cm × 5.5 cm, with poorly defined margins (Figure 1). The cortex of the caudal mediastinal lymph node was effaced by multinodular nodules, with similar features as those described in the left lung lobe.

**Figure 2:**
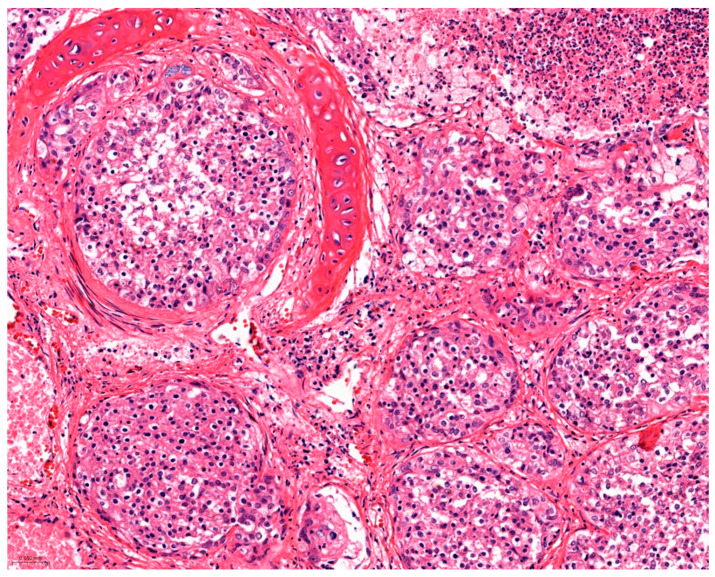
Histological lung section showing neoplastic proliferation filling bronchi and alveoli lumen, H&E, 20×.

Histological examination of the lung revealed a densely cellular, poorly demarcated, encapsulated, infiltrative, multifocal to coalescent epithelial neoplasm filling the lumina of alveoli, bronchioles, and bronchi. Neoplastic cells were arranged in solid packets (Figure 2), which were loosely packed and supported by a delicate fibrovascular stroma. Neoplastic cells were moderately differentiated, cylindrical to cuboidal, with indistinct cell borders, large amounts eosinophilic and occasionally finely vacuolated cytoplasm, with a central round to oval nucleus showing, with stippled chromatin and indistinct nucleoli. Nuclear pleomorphism was mild with overall uniform nuclei and minimal anisocytosis and anysokaryosis. Mitotic count was four in 2.37 mm^2^ [18]. There were multifocal areas of tumor necrosis [21% to 50%] and tumor fibrosis [1% to 20%] [19]. Furthermore, lymphovascular invasion, multiple foci of squamous metaplasia, and multifocal mineralization, possibly compatible with degenerated parasitic structures, were also observed in the neoplastic mass.

**Figure 3:**
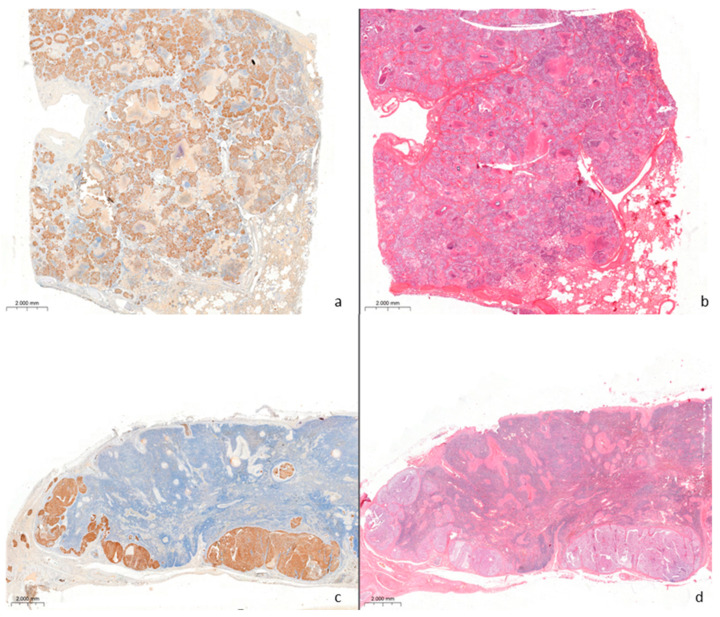
Histological and immunohistochemical findings in the lung and caudal mediastinal lymph node with a bronchioloalveolar carcinoma (4×). (**a**) Immunohistochemical positivity to CKAE1/AE3 in neoplastic cells. (**b**) The same lung section H&E stained. (**c**) Immunohistochemical positivity to CKAE1/AE3 in the metastatic site of lymph node. (**d**) The same lymph node section H&E stained showing subcapsular metastasis.

Areas of neoplastic proliferation were surrounded by fibrovascular stroma and growths of fibroblasts and inflammatory lymphocytic infiltrates. The caudal mediastinal lymph node displayed multifocal to coalescent, metastatic nodules infiltrating the capsule, the cortex, and partially the medulla. Neoplastic cells showed strong cytoplasmic and membranous immunoreactivity to pan-cytokeratin AE1/AE3 (Figure 3a–d) and mild cytoplasmic immunoreactivity to cytokeratin 7. The neoplastic cells showed no immunoreaction for high molecular weight cytokeratin and vimentin.

Additional histopathological findings include severe diffuse acute suppurative pyelonephritis associated with nephrolithiasis, adult gastric helminths consistent with nematodes, and moderate multifocal chronic lymphoplasmacytic interstitial orchitis.

## 4. Discussion

Most reported neoplasm cases were incidental findings during the autopsy of stranded animals. Species belonging to the delphinidae family were the aquatic mammals in which neoplastic pathologies were described most frequently [3]. Although spontaneous neoplasms were single and sporadic reports in different species, the increased incidence of neoplasia in the same population could suggest the presence of exposure factors like pollutants and anthropogenic activity. Correlation between cancer and contaminated aquatic ecosystems is well known, as demonstrated by the discovery of numerous specimens of beluga whales affected by intestinal adenocarcinoma in the Saint Lawrence Estuary where the aquatic habitat was extremely polluted due to aluminum effluents [20].

Numerous studies link the onset of cancer to environmental pollution and some of these suggest specific physiological mechanisms such as dysbiosis and immune dysfunction [21]. Contaminants known to have oncogenic effects include polycyclic aromatic hydrocarbons (PAHs), dioxins, polychlorinated biphenyls (PCBs), pesticides such as dichlorodiphenyltrichloroethane (DDT), and heavy metals [21]. Their role is crucial as exposure factors for the onset of cancer, but the physiological mechanism still requires further investigation.

Among the most frequent neoplasms, the epithelial tumors were represented with a variable number between benign and malignant ones. This paper described the first report of a lung neoplasia in a striped dolphin. The morphological characteristics, growth pattern of the neoplastic cells, and immunohistochemical analysis allowed the pathologist to identify the tumor as bronchioloalveolar carcinoma with lymph node metastatic dissemination. The neoplasm was considered as primary in the lung, excluding the possibility that it could be a secondary form due to the macroscopic aspect of the lesion described as focal and not circumscribed and also to the absence of significant neoplastic lesions in other sites. Despite the marked cellular atypia observed, immunohistochemical examination revealed the negativity of the neoplastic tissue areas to vimentin and the positivity to cytokeratins AE1/AE3 and CK7, confirming the epithelial origin of the tumor.

## Data Availability

The original contributions presented in this study are included in the article. Further inquiries can be directed to the corresponding author.
